# Epidemiological trends of respiratory tract pathogens detected via mPCR in Australian adult patients before COVID-19

**DOI:** 10.1186/s12879-023-08750-7

**Published:** 2024-01-02

**Authors:** Audrey K. Grech, Chuan T. Foo, Eldho Paul, Ar K. Aung, Christiaan Yu

**Affiliations:** 1 Department of Respiratory Medicine, Alfred Health, 55 Commercial Road, Melbourne, VIC 3004 Australia; 2 Department of Respiratory Medicine, Eastern Health, Melbourne, Australia; 3 Monash Lung and Sleep, Monash Health, Melbourne, Australia; 4 ANZIC-RC, School of Public Health and Preventive Medicine, Monash University, Melbourne, Australia; 5 School of Public Health and Preventive Medicine, Monash University, Melbourne, Australia; 6 Department of General Medicine, Alfred Health, Melbourne, Australia; 7 Central Clinical School, Monash University, Melbourne, Australia

**Keywords:** Respiratory tract infections, Seasonal distribution of respiratory pathogens, Viral trends

## Abstract

**Background:**

Respiratory tract infections (RTIs) are a major global health burden due to their high morbidity and mortality. This retrospective study described the epidemiology of respiratory pathogens in adults over a 5-year period at an Australian tertiary healthcare network.

**Methods:**

All multiplex reverse transcription polymerase chain reaction respiratory samples taken between the 1st of November 2014 and the 31st of October 2019 were included in this study. Overall prevalence and variations according to seasons, age groups and sex were analysed, as well as factors associated with prolonged hospital and intensive care length of stay.

**Results:**

There were 12,453 pathogens detected amongst the 12,185 positive samples, with coinfection rates of 3.7%. *Picornavirus (Rhinovirus*), Influenza A and respiratory syncytial virus were the most commonly detected pathogens. *Mycoplasma pneumoniae* was the most commonly detected atypical bacteria. Significant differences in the prevalence of *Chlamydia pneumoniae* and *Human metapneumovirus* infections were found between sexes. Longest median length of intensive care and hospital stay was for *Legionella* species. Seasonal variations were evident for certain pathogens.

**Conclusions:**

The high rates of pathogen detection and hospitalisation in this real-world study highlights the significant burden of RTIs, and the urgent need for an improved understanding of the pathogenicity as well as preventative and treatment options of RTIs.

## Summary

Distinct trends of respiratory pathogens observed across seasons, age groups and sex contribute to a large burden of disease during the pre-COVID-19 era. Of viral pathogens, *Picornavirus*, influenza A and respiratory syncytial virus were the most common viral pathogens whilst *Mycoplasma pneumoniae* was the most common atypical bacterial pathogen detected on multiplex reverse transcription polymerase chain reaction.

## Introduction

Respiratory tract infections (RTIs) are a major global health burden due to their high morbidity and mortality. They accounted for approximately 336.5 million infections and 2.4 million deaths in a 2016 global burden of disease estimate [1, 2]. Although most RTIs are self-limiting, they can contribute to severe outcomes including hospitalisation and death [1]. This is particularly seen in young children, the elderly and those who are immunocompromised [3, 4]. In Australia, RTIs account for up to 7 million visits to general practitioners each year, with an average individual experiencing 2 to 5 episodes of infection per year [5]. Due to their high prevalence, RTIs place an enormous burden on healthcare systems, imposing a significant economic cost in terms of direct medical expenses including hospitalisation, primary care reviews and antibiotic prescriptions as well as indirect productivity losses [6].

The majority of RTIs are caused by viruses, followed by bacterial infections [7, 8]. Due to the undifferentiated nature of respiratory symptoms at presentation between pathogen type/s, diagnostic uncertainty contributes to the mounting costs of investigations and antibiotic overprescribing [9]. Multiplex reverse transcription polymerase chain reaction (mPCR) allows the rapid detection of a panel of respiratory pathogens (inclusive of both viruses and bacteria) and identifies coinfection in a single test [10, 11]. mPCR has largely replaced previous testing methods, including cultures, which were less sensitive, have limited ability for pathogen detection and are more time consuming. Whilst mPCR allows for more tailored treatment for patients, on a larger scale, it is also useful in the monitoring of epidemiological trends of RTIs [12].

In recent times, interest in respiratory pathogens has increased due to the emergence of several novel viruses. These include severe acute respiratory syndrome coronavirus 2 (SARS-CoV-2), which causes COVID-19 illness and influenza A virus subtype H5N1 [12, 13]. Up to 30% of adults admitted to hospital with community acquired pneumonia (CAP) prior to the COVID-19 pandemic had a viral aetiology [14], with an overall mortality of 3% [15] and over 15–20% of severe CAP cases in adults were attributed to influenza alone [16]. Furthermore, respiratory syncytial virus (RSV) is increasingly being recognized as a cause of illness in high-risk adults, including those with chronic cardiorespiratory disease, and those who are immunocompromised [17] or older [18]. Such patient groups are particularly at risk of severe infection, with intensive care unit (ICU) admissions and mortality rates being similar to that of influenza [19, 20]. Currently, available vaccines only target against *Streptococcus pneumoniae*, *Haemophilus influenzae* B, influenza A and B, and COVID-19.

Both influenza and pneumococcal epidemiology is well defined through surveillance systems [21–23]. The influenza season is known to coincide with the cooler months in Australia, from May to October each year (end of autumn to mid-spring), with the remaining six months defined as non-influenza season [24]. However, seasonal variations of other respiratory pathogens are less studied in the temperate southern hemisphere. An improved understanding of the epidemiology of RTIs pathogens may help manage seasonal outbreaks and individual patients more effectively. Furthermore, variations according to host factors, including age and sex, are not well understood in the Australian context, nor are the impacts of RTIs on healthcare resource utilisation such as hospital length of stay (LOS) in either internal medicine wards (IMWs) or ICUs.

The objective of this retrospective study was to describe the overall prevalence of non-pneumococcal respiratory pathogens in Australian adults at a single healthcare network between 2014 to 2019, and identify variations in season, sex and age that were associated with ICU admission and IMW LOS. Considering the paucity of Australian data on RTI epidemiology and pathogens, this information may assist in bettering the understanding of outbreaks as well as healthcare resource allocation.

## Methods

Data for this retrospective study was collected across 3 of Monash Health’s acute hospitals in metropolitan Melbourne, Australia. Monash Health is a public health network that serves as a catchment for over one quarter of the state’s 6.6 million residents and has 1,389 beds across the 3 sites. There are 20 acute public hospitals in Metropolitan Melbourne, with 10 having ICUs. Most of the healthcare in Australia is provided in a public setting with less than 30% of patients optionally presenting to private hospitals [25]. In Australia, all citizens and permanent residents can receive universal health coverage under the Medicare scheme. This includes access to mPCR testing, as indicated by the presence of respiratory tract infection symptoms in those receiving care in a hospital setting. Data from all adult patients with a respiratory mPCR result dated between the 1st of November 2014 and the 31st of October 2019 were included in the study. Samples were obtained by the treating physicians when clinical indications (e.g. respiratory symptoms, systemic symptoms, radiographic changes, etc.) were present on presentation to ED or within 48 h of inpatient admission. Samples were collected from the upper and/or lower respiratory tracts. Upper respiratory tract samples were defined as any sample taken from the nose, throat or nasopharynx using standardised nasopharyngeal swab procedure across Monash Health’s sites. Lower respiratory tract samples included spontaneously expectorated sputum, endotracheal or bronchoscopic specimens. Samples were processed at the microbiological laboratory onsite at the hospital it was collected at; Casey Hospital, Dandenong Hospital or Monash Hospital, respectively. Samples were processed using AusDiagnostics High-Plex Respiratory Pathogens Assay (New South Wales, Australia) according to the manufacturer’s instructions. The assay was used to detect the following 9 viruses and 4 atypical bacteria: *Adenovirus, Human metapneumovirus* (HMPV), influenza A and B, parainfluenza virus 1, 2 and 3 (PIV 1, 2 and 3), *Picornavirus*, RSV, *Bordetella spp*, *Chlamydia pneumoniae*, *Legionella spp*, and *Mycoplasma pneumoniae*. The assay was unable to detect *Bordetella spp. and Legionella spp* until after 2015 or distinguish *Rhinovirus* from *Enterovirus*; hence these were grouped together as *Picornaviruses*. For this study, we assumed that almost all *Picornaviruses* were *Rhinoviruses* given the clinical context. Data was retrospectively retrieved from patient files via the Monash Health electronic medical records system.

### Statistical analysis

The distribution of respiratory pathogens was analysed by age, sex and season. The four seasons in the southern hemisphere are: summer (December-February), autumn (March-May), winter (June-August) and spring (September-November). The age of patients was stratified into 3 categories as is common in clinical practice: 18–20 years, 20–70 years, and elderly patients, as per the ASPREE study’s definition being > 70 years [21]. LOS data was categorised by virus type as well as by nature of hospital admission. Duplicate samples were defined as positive samples taken from the same patient within a 30-day period from the initial positive test and were not included in the analysis. Since the samples were obtained from a single healthcare network, site specific distribution of samples was not analysed.

Categorical variables were presented as counts and proportions. Continuous variables were assessed for normality and summarised using mean and standard deviation (SD) or median and interquartile range (IQR) according to data type and distribution. Group comparisons were performed using chi-square test for equal proportions or Fisher’s exact test where numbers were small. A two-sided *p* value < 0.05 was considered statistically significant. Statistical analysis was performed using the SAS software version 9.4 (SAS Institute, Cary, NC, USA).

## Results

### Patient demographics

**Fig. 1:**
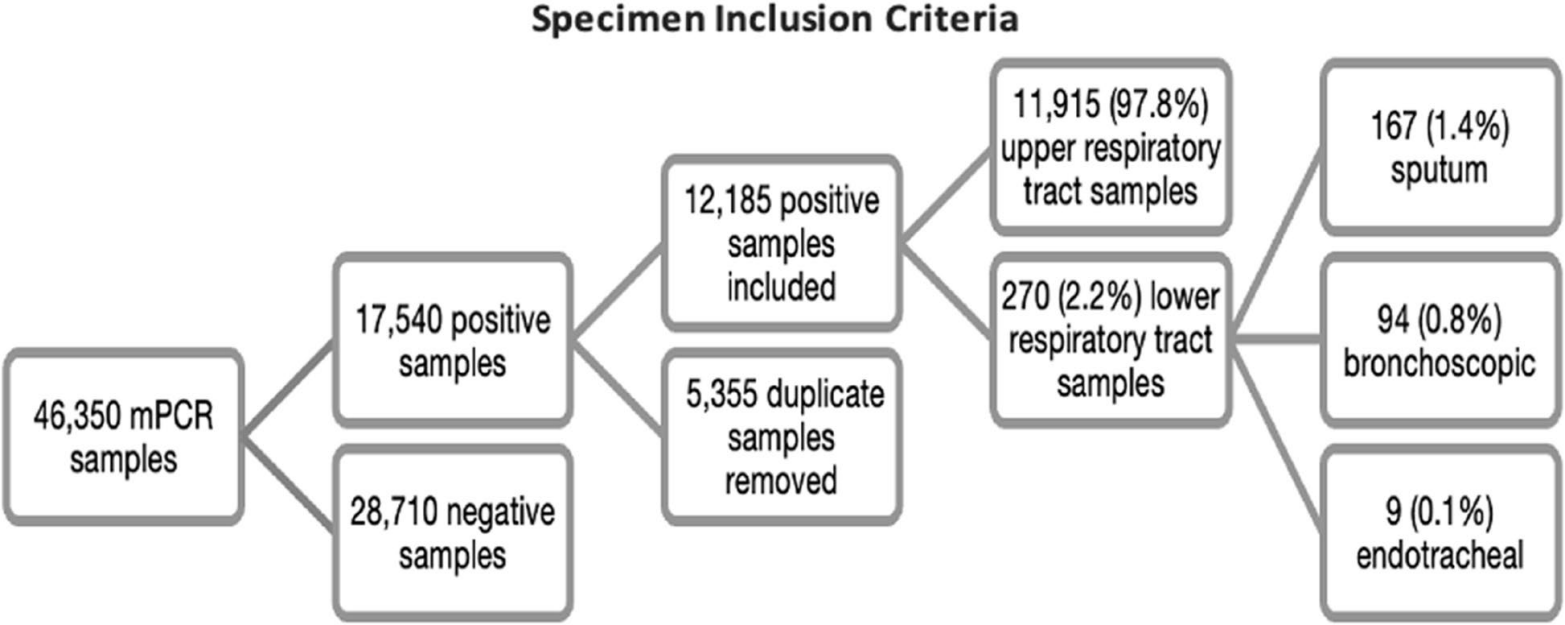
Number of mPCR samples, including exclusion data and location of each sample type

Figure 1 depicts sample size and specimen origin data. Of the 46,350 samples, 17,540 were positive for at least one pathogen. Once duplicates were removed from the dataset, there were 12,185 (29.7%) samples that were suitable for inclusion in the data analysis. The mean (SD) age of patients with a positive sample was 62.0 (21.2) years, and 6,408 patients (52.6%) were female. The frequency of pathogens across both sexes is displayed in Table 1. Statistically significant differences in RTI frequency was seen between the sexes for *HMPV* and *C. pneumoniae with more cases being seen in females (HMPV 9.2% vs. 7.6%, p = 0.002) and males (C. pneumoniae 0.8% vs. 0.5%, p = 0.006), respectively.*

**Table 1:** Pathogen distribution by Sex (n, %)

Pathogen	All patients (*n* = 12,185)	Female (*n* = 6,408)	Male (*n* = 5,777)	*P* Value
***Picornavirus***	4008 (32.9)	2075 (32.4)	1933 (33.5)	0.21
**Influenza A**	3467 (28.5)	1794 (28.0)	1673 (29.0)	0.24
**RSV**	1353 (11.1)	709 (11.1)	644 (11.1)	0.88
***HMPV***	1024 (8.4)	587 (9.2)	437 (7.6)	0.002
**Influenza B**	1092 (9.0)	581 (9.1)	511 (8.8)	0.67
**PIV-3**	791 (6.5)	442 (6.9)	349 (6.0)	0.055
***Adenovirus***	253 (2.1)	133 (2.1)	120 (1.2)	0.99
**PIV-1**	135 (1.1)	74 (1.2)	61 (1.1)	0.60
**PIV-2**	85 (0.7)	47 (0.7)	38 (0.7)	0.62
***M. pneumoniae***	92 (0.8)	46 (0.7)	46 (0.8)	0.62
***Bordetella Spp.***	58 (0.5)	32 (0.5)	26 (0.5)	0.69
*** C. pneumoniae***	78 (0.6)	29 (0.5)	49 (0.8)	0.006
***Legionella Spp.***	17 (0.1)	5 (0.1)	12 (0.2)	0.086

### Prevalence of respiratory pathogens

Across the 12,185 positive samples, a total of 12,453 pathogens were identified (12,208 [98%] viruses and 245 [2%] bacteria). The prevalence of respiratory pathogens across the study period is presented in Table 1, with Picornavirus (32.9%), Influenza A (28.7%) and RSV (11.1%) being the most prevalent. *M. pneumoniae* was the most common atypical bacteria detected (0.8%).

### Respiratory pathogen co-infection

The singular and co-infection rates are outlined in Table 2, with a breakdown of viral versus bacterial aetiology. The three most common viruses detected in dual-positive samples were *Picornavirus* (48.6%), influenza A/B (47.3%), and RSV (24%). In triple-positive samples, these were influenza A/B (90%), RSV (40%) and *C. pneumoniae* (30%).

**Table 2:** Pathogen type in single and co-infection

Pathogen type	Virus, n (%)	Bacteria, n (%)	All pathogens, n (%)
**Single**	11,533 (98.3)	200 (1.7)	11,733 (96.3)
**Dual**	400 (90.5)	42 (9.5)	442 (3.6)
**Triple**	7 (70)	3 (30)	10 (0.1)

Dual infections were seen in all age groups, with almost a third occurring in individuals > 70 years old. In contrast, triple infections were only detected in 10% of individuals > 70 years, with the majority (50%) found in those over 40 years of age.

### Sample collection location

Of the 12,185 samples, 8,876 samples were collected in ED, 797 in ICU and the remaining 2,512 on IMWs.

### Relationship between respiratory pathogens and age

The prevalence of each pathogen type across the three categories of age is shown in Table 3.

**Table 3:** Pathogen prevalence by age group (n, %)

Pathogen	18–20 years (*n* = 194)	20–70 years (*n* = 6,677)	> 70 years (*n* = 5314)	*P* value
***Picornavirus***	85 (43.8)	2405 (36.0)	1518 (28.6)	< 0.0001
**Influenza A**	56 (28.9)	1813 (27.2)	1598 (30.1)	0.002
**Influenza B**	18 (9.3)	688 (10.3)	386 (7.3)	< 0.0001
**RSV**	11 (5.7)	603 (9.0)	739 (13.9)	< 0.0001
**HMPV**	8 (4.1)	491 (7.4)	525 (9.9)	< 0.0001
**PIV-3**	2 (1.0)	355 (5.3)	434 (8.2)	< 0.0001
***Adenovirus***	7 (3.6)	187 (2.8)	59 (1.1)	< 0.0001
***M. pneumoniae***	4 (2.1)	82 (1.2)	6 (0.1)	< 0.0001
**PIV-1**	1 (0.5)	62 (0.9)	72 (1.4)	0.067
*** C. pneumoniae***	3 (1.5)	60 (0.9)	15 (0.3)	< 0.0001
**PIV-2**	0 (0.0)	48 (0.7)	37 (0.7)	0.78
***Bordetella spp.***	1 (0.5)	37 (0.6)	20 (0.4)	0.38
***Legionella spp.***	0 (0.0)	15 (0.2)	2 (0.04)	0.019

### Seasonal distribution of different respiratory pathogens

**Fig. 2:**
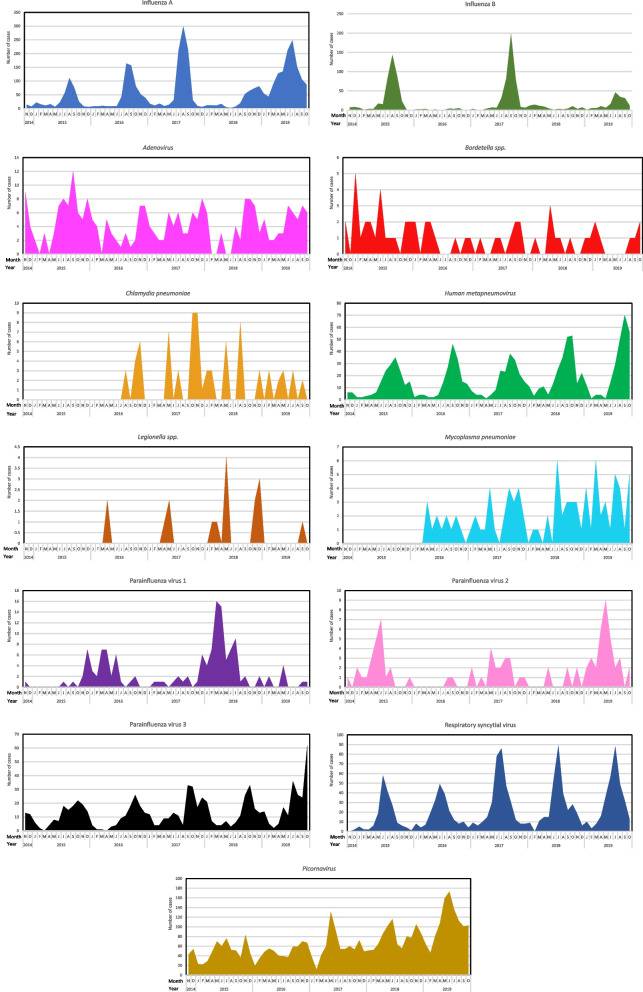
Seasonal variation in respiratory pathogen prevalence by year. Summer: December to February, Autumn: March to May, Winter: June to August and Spring: September to November

Seasonal variations for respiratory pathogens studied are shown in Fig. 2. While the majority of pathogens followed distinct seasonal patterns, the trends of the three most common pathogens (*Picornavirus*, influenza A, and RSV) are as follows. *Picornavirus* circulated all year round, with bimodal peaks in autumn and late spring, and a trough in mid-late summer. Influenza A showed seasonal peaks in late winter and early spring and had a similar number of positive samples to *Picornavirus* in spring. Lastly, RSV showed a seasonal pattern, with the highest number of presentations being in early winter.

### Hospitalisation duration for respiratory pathogens

Ten thousand three hundred twenty-six (86.5%) patients were managed on internal medicine wards (IMWs), and 797 (6.7%) in ICUs. *Picornavirus* (IMW *n* = 3383, ICU *n* = 320), influenza A (IMW *n* = 2788, ICU *n* = 167) and RSV (IMW *n* = 1160, ICU *n* = 86), respectively, accounted for the most hospital admissions. Median LOS for ED, ICU and IMW hospitalisation according to different RTI pathogens are presented in Table 4. The median length of stay in each department accounting for all pathogens was 7.1 h in ED, 4.5 days in ICU and 3.6 days in IMWs.

**Table 4:** Hospital and ICU length of stay by pathogen

Pathogen		ICU length of stay (days)		Hospital length of stay (days)	
		n	Median (IQR)	n	Median (IQR)
***Picornavirus***	320		2.8 (1.5–5.2)	3383	3.5 (1.7–7.3)
**Influenza A**	167		2.7 (1.6–5.2)	2788	3.0 (0.8–6.9)
**Influenza B**	48		4.0 (1.9–6.9)	855	2.3 (0.5-5.0)
**PIV-1**	14		6.4 (1.9–12.6)	120	4.1 (2.2-9.0)
**PIV-2**	2		7.4 (1.8–13.0)	66	3.4 (1.8–7.7)
**PIV-3**	60		3.6 (1.9–6.7)	690	4.0 (1.9–9.7)
**RSV**	86		3.6 (1.9–6.2)	1160	4.0 (2.0–9.0)
**HMPV**	57		3.9 (2.6–6.5)	849	3.7 (1.8-7.0)
***Adenovirus***	25		4.2 (2.1–5.8)	212	2.9 (0.8–5.7)
***M. pneumoniae***	2		1.7 (1.7–1.7)	73	2.8 (0.9–4.7)
***Legionella Spp.***	5		8.8 (8.5–24.7)	17	6.8 (5.5–15.0)
***Bordetella Spp.***	1		5.9 (5.9)	44	2.7 (1.0-5.7)
***C. pneumoniae***	10		3.2 (2.3–7.8)	69	4.0 (1.9–6.8)

## Discussion

To date, there have been few large-scale Australian studies examining the epidemiology of respiratory pathogens in individuals presenting to acute hospitals. In this retrospective study, we found a modest positivity rate of 29.7% for at least one respiratory pathogen. Our results are slightly lower than the 30–45% positivity rate reported by other studies that utilised mPCR testing of nasopharyngeal samples [5, 22]. Our lower detection rate may relate to excluding duplicate positive results for the same individual, as well as inclusion of lower respiratory tract samples, while other studies did not.

Overall, *Picornavirus* (predominantly *Rhinovirus*) was the most frequently detected pathogen. This particularly affected the 20–70 year old age group, which is consistent with findings of previous publications [24, 26]. Previously, picornaviruses were thought to represent a mild disease, however there is now increasing evidence that picornaviruses play an important role in exacerbations of chronic respiratory diseases and contribute to morbidity and mortality [27]. Recent data also suggest an increasing association between *Picornavirus* and severe hospital and community acquired pneumonia [9, 28], with one study reporting higher mortality in adults hospitalised with rhinovirus when compared with influenza [29]. In our study, not only was *Picornavirus* the most prevalent RTI pathogen, it also contributed to the highest hospitalisation rate, thus reflecting its importance as an emerging health threat and highlighting the need for urgent action, given there are limited vaccine and therapeutic options against it.

Influenza A was found to be the second most common RTI pathogen. It demonstrated the expected seasonal pattern of having highest case numbers in winter and spring. This seasonality has been reflected in temperate regions around the world, while tropical regions tend to have a more diverse outbreak pattern [30]. In our cohort, influenza A had one of the shortest ICU LOSs, and a modest LOS in IMWs, despite being the second most common pathogen causing hospitalisation. Although previous literature is scarce, it notes that the average duration of hospitalisation for influenza is between 6.5 and 8.3 days [31] and that 6% of patients require care in the ICU [32]. This is similar to our rate of 5.9%. In one of the only prospective studies in this field, Thompson et al. determined that there is a reduction in ICU LOS and a 59% reduction in the risk of an ICU stay for those that are vaccinated for influenza [33]. Under the Australian National Immunisation Program, it is estimated that up to 70% of those aged above 75 years receive the influenza vaccine annually [34]. However, despite the eligibility criteria for influenza vaccination at no cost, vaccine uptake by patients with ‘at-risk’ conditions remains variable, thus opportunities still exist to improve vaccination rates to lessen the disease burden [35].

We observed RSV to be the third most prevalent virus in our study, with peak numbers in autumn and winter. As expected, higher positivity rates were seen in those > 70 years old compared to younger age groups in this study. Commonly associated with bronchiolitis and pneumonia in children, RSV is now recognised as an important pathogen in adults, especially in the elderly and those with comorbid cardiorespiratory disease [36]. Severe complications such as respiratory failure, prolonged hospitalisation and mortality rates similar to seasonal influenza have been observed in adults admitted to hospital with RSV infections. Interestingly, the risk of death has been reported to be higher from RSV than influenza after adjusting for comorbidities [37]. Our data demonstrates that the median LOS for IMWs and ICU for RSV was higher than influenza A. Although the use of RSV-specific immunoglobulins, palivizumab and ribavirin, have been studied in infants, there is limited data regarding their efficacy in adults, with the mainstay of treatment being symptomatic management [16]. Given the frequency of RSV detection in hospitalised patients and the poor outcomes for certain patient groups, the unmet need for antiviral and immunoglobulin therapy and vaccination against RSV in adults should be promptly explored. Furthermore, exploring the efficacy of inpatient isolation is a simple measure that may be highly effective in the containment of cases.

*M. pneumoniae* was a common cause of respiratory tract infections before the COVID-19 pandemic, with worldwide incidence of 8.6% from 2017 to 2020, measured by direct test methods [38]. The incidence reduced to 1.7% between 2020 and 2021 [38]. A study by Sauteur et al. (2023) comments on the scarcity of *M. pneumoniae* in the post-pandemic world, which is thought to be a result of the non-pharmaceutical interventions utilised to prevent COVID-19 transmission. We note that in our pre-COVID pandemic dataset, *M. pneumoniae* was the most commonly detected atypical bacteria. Thus, it is important for future Australian studies to monitor the prevalence of the disease, with particular attention being paid to the possibility of resurgence in a population potentially lacking immunity.

Our co-infection rate of 3.7% was higher than the results reported by Vissaeux et al. who found an incidence of 1.6% in 7,196 samples over 5 years [12]. A reason for this could lie in geographic differences and host factors such as immunosuppression which we did not explore in this study. Interestingly, within the constraints of the bacterial infections that we tested for via mPCR, we found that the majority of coinfections in our study included viruses only, with few coinfections including bacterial pathogens. Previous studies suggest that some viruses, particularly rhinoviruses, are able to reduce the ability of other viruses to establish infection via viral-viral interference, whilst the opposite is true for other pathogens [39–41]. This would suggest that there would be less viral coinfections. However, the difference in results may be explained by the limited number of bacterial pathogens that were possible to be detected via our mPCR panel.

Ultimately, our study has several limitations. Firstly, the cohort sampled was those who presented or were referred to a tertiary hospital for any cause. Such referral bias may not be a true reflection of a random sample of the broader Australian community. Secondly, we did not explore the clinical data, hence, a more accurate estimation of LOS directly attributable to individual pathogens was not possible. Additionally, risk factors such as immunocompromised status may further contribute to the aetiology of infection and LOS. Lastly, our mPCR assay did not detect other respiratory viruses such as coronavirus and bocavirus, was incapable of distinguishing enterovirus from rhinovirus as well as the H1N1 and H5N1 strains of influenza A, and we did not include various other bacterial infections such as pneumococcus or those that could be detected/isolated by antigens, microscopy and cultures in our data collection. Consequently, any differential distribution or pathogenicity could not be assessed, and our co-infection prevalence may be underreported.

Since the introduction of public health measures, which include social distancing and mask wearing to curb the spread of COVID-19 between 2019 to 2022, it has been noted that there has been a major decrease in the prevalence of all respiratory viral infections [42, 43]. On a global scale, influenza circulation was particularly low in 2020 as was demonstrated by near complete elimination in Taiwan [44] and significantly lower case numbers in various other countries [21]. Whilst a resurgence started in late 2021, in the first part of 2022 with easing of some of public health measures, seasonal patterns had not returned to normal in the Northern or Southern hemisphere [23]. Over the coming months and years, it will be an imperative to monitor if trends for both influenza and other pathogens return to that of something similar to pre-pandemic times, or if new patterns of RTI pathogens arise, especially in relation to climate change.

In summary, we have provided a broad snapshot of the epidemiology and healthcare utilisation for patients presenting to hospital with acute RTIs over a five-year period in Melbourne, Australia. The high rates of non-pneumococcal aetiology of RTI positivity obtained in this real-world study highlights the significant burden of infection, dominated by *Picornavirus*, influenza A and RSV, and the urgent need for preventative and treatment options. Furthermore, in seeing patterns of RTIs and the burden they place on the healthcare system, funding can be guided towards appropriate measures to improve healthcare utilisation, as well as reduce spread of disease, mortality and morbidity. In turn, this will prevent loss of productivity, improve individuals’ health and quality of life, and decrease healthcare expenditure. Future research is needed to improve our understanding of the pathogenicity of these viruses and note new trends in epidemiology post COVID-19 lockdowns and due to impact of climate change.

## Data Availability

The datasets used and/or analysed during the current study available from the corresponding author on reasonable request.
